# Hepatobiliary Complications Associated With Ketamine Use: Clinical Insights and Future Directions

**DOI:** 10.7759/cureus.84974

**Published:** 2025-05-28

**Authors:** Hafiza Wajeeha Waheed, Muhammad R Ashraf, Talha Sajjad, Hanzala Jehangir, Iqra Baig, Ahmad Zulaid, Abdul Rehman Nasir, Allah Dad

**Affiliations:** 1 Emergency Medicine, Maimonides Medical Center, New York, USA; 2 Acute Medicine, The Princess Alexandra Hospital NHS Trust, Harlow, GBR; 3 Internal Medicine, Sheikh Zayed Medical College and Hospital, Rahim Yar Khan, PAK; 4 Internal Medicine, Bahawal Victoria Hospital, Bahawalpur, PAK; 5 Internal Medicine, Quaid-e-Azam Medical College, Bahawalpur, PAK

**Keywords:** biliary duct abnormalities, chronic ketamine user, drug-induced hepatotoxicity, drug-induced liver failure, liver fibrosis, side effects of ketamine

## Abstract

Ketamine has been utilized in various medical contexts, particularly for its effects on the nervous system. Over time, its applications have expanded beyond its initial use, with emerging interest in its potential to influence mood and cognition. In certain clinical populations, it has been associated with a rapid reduction in depressive symptoms and suicidal thoughts, particularly when conventional treatments have proven insufficient. Despite these promising therapeutic effects, concerns remain regarding possible adverse consequences, including those related to liver and biliary function. This review explores the current understanding of how ketamine use may impact hepatic and biliary health, with a focus on observed complications, possible underlying mechanisms, clinical manifestations, and available management strategies.

A structured examination of existing medical literature was conducted, drawing from multiple scientific sources to identify research on ketamine’s effects on liver and biliary function. Studies involving human participants were included, particularly those documenting changes in liver enzyme levels, disruptions in bile flow, and structural alterations in the biliary system. The selection criteria emphasized original investigations, case reports, and clinical evaluations published in peer-reviewed sources.

Findings indicate that ketamine exposure has been associated with a spectrum of liver and biliary changes, ranging from mild laboratory abnormalities to more serious conditions that require medical intervention. Several possible biological mechanisms have been proposed, including effects on bile flow regulation, oxidative stress, and toxicity affecting liver function. Notably, discontinuing ketamine use has been linked to improvements in many cases, and various treatment approaches, including supportive care and specific pharmacologic interventions, have been explored to alleviate symptoms.

As ketamine continues to be incorporated into diverse therapeutic settings, understanding its full range of effects remains crucial. Further research is needed to clarify how these biological changes occur, identify individuals who may be more susceptible, and develop strategies to ensure safer use across different clinical and nonclinical populations.

## Introduction and background

Ketamine is a glutamate N-methyl-D-aspartate receptor (NMDA-R) antagonist that has been clinically used since the 1960s, primarily as an anesthetic. Chemically, it consists of two enantiomers, R-ketamine and S-ketamine, the latter having a higher affinity to NMDA-R [1]. Ketamine is mostly administered intravenously, but it can also be administered subcutaneously, intramuscularly, transdermal, intranasally, intrarectally, or orally [2]. It acts primarily through glutamate modulation, affecting NMDA and AMPA receptors, and activating BDNF and mTOR signaling pathways [3]. Ketamine's unique pharmacology allows it to maintain cardiorespiratory stability while providing effective sedation and analgesia [4]. In recent years, ketamine has shown promise as a rapid-acting antidepressant, particularly for treatment-resistant depression and potentially for reducing suicidal ideation [3]. While ketamine has shown promise for its antidepressant effects and potential application in other areas, its widespread use remains limited due to side effects and risk of abuse [5,6]. Common acute effects include dissociation, nausea, dizziness, sedation, and cardiovascular stimulation [7,8]. Long-term use or high doses can lead to neurotoxicity, cognitive dysfunction, and uropathic effects [9]. The incidence and severity of these side effects can vary based on factors like age, gender, and dosage [10]. Among these adverse effects, ketamine’s impact on the hepatobiliary system is of particular concern, with reports of liver enzyme elevation and cholestasis [11]. Given the increasing use of ketamine in both clinical and nonclinical settings, understanding its potential hepatobiliary toxicity is crucial for patient safety and informed clinical decision-making. More studies are needed to explore such potential side effects as well as mitigation strategies to reduce side effects and increase tolerability. This literature review aims to evaluate the current evidence on ketamine-associated hepatobiliary side effects, focusing on their prevalence, underlying mechanisms, clinical presentations, and possible management strategies.

## Review

Methods

**Figure 1:**
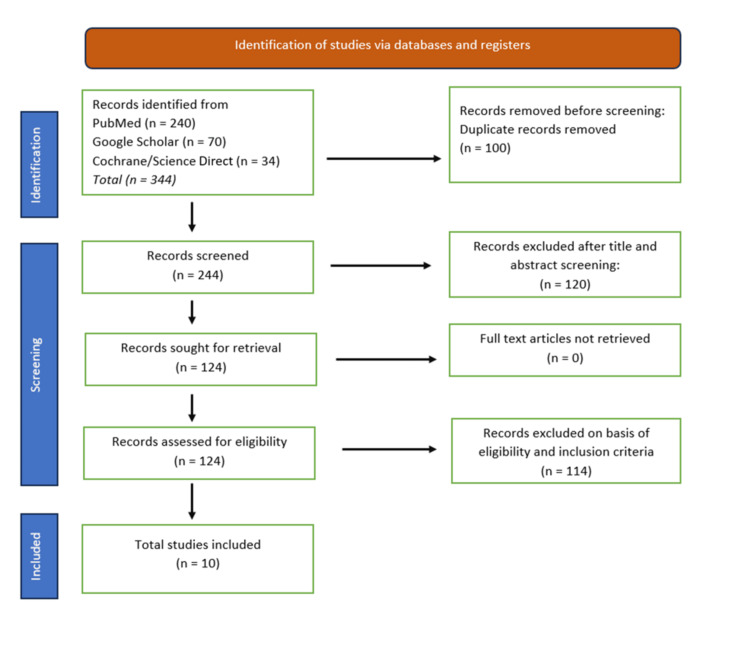
Flowchart depicting the study selection process for the scoping review

A comprehensive literature search was conducted using multiple databases, including PubMed, Google Scholar, Science Direct, and Cochrane Library, to identify relevant studies on ketamine-associated hepatobiliary side effects. The search strategy employed keywords such as "ketamine", "hepatobiliary side effects", "liver injury", "cholestasis", and "liver enzymes" to capture all potential articles. The initial search yielded 344 articles. These were screened based on their titles and abstracts, and after applying the inclusion and exclusion criteria, 10 articles were selected for full-text review and inclusion in the final analysis. The selected articles were reviewed to assess the limitations reported by the authors. A narrative synthesis approach was used to analyze and summarize the findings from the selected studies, with an emphasis on identifying patterns, prevalence, mechanisms, and clinical implications of ketamine-induced hepatobiliary side effects. The complete search strategy and selection of studies are depicted in the research flowchart shown in Figure 1.

Eligibility Criteria 

The inclusion criteria for this review focused on studies involving human subjects to ensure direct clinical relevance, specifically targeting articles that examined hepatobiliary side effects or hepatotoxicity associated with ketamine use, including liver enzyme elevation, cholestasis, biliary tract abnormalities, or liver failure. Only original research articles, case reports, case series, clinical trials, cohort studies, and retrospective analyses were considered, provided they were peer-reviewed to uphold a high standard of scientific rigor. Additionally, studies had to be published in English to maintain accessibility and consistency in data interpretation. Conversely, the exclusion criteria eliminated studies involving nonhuman subjects, unless they provided significant mechanistic insights applicable to humans. Research not specifically addressing hepatobiliary side effects or hepatotoxicity related to ketamine use was excluded, along with studies involving multi-drug-induced injuries or polypharmacy, where the role of ketamine could not be isolated. Reviews, editorials, opinion pieces, and studies lacking original data or quantitative analysis were also excluded. Furthermore, studies with insufficient data, unclear methodologies, or non-peer-reviewed sources were not included, and articles in languages other than English were excluded to prevent translation bias and ensure consistent interpretation of results. 

Results

Our literature indicates that a growing body of evidence suggests that ketamine, while therapeutically valuable, poses risks of significant hepatobiliary complications. The studies reviewed highlight various aspects of ketamine-associated liver and biliary tract injuries, shedding light on the clinical manifestations, underlying mechanisms, and potential outcomes. A systematic review comprehensively examined 11 cases of ketamine-associated biliary disease, providing one of the most robust assessments of this issue to date [12]. The cases primarily involved young males, with a mean age of 25 years, and the median duration of ketamine abuse was 24 months. The most common clinical presentation was abdominal pain in 71% of patients, often accompanied by elevated liver enzymes and biliary tract abnormalities. Expanding on these clinical presentations, a compelling case study described a middle-aged man with chronic ketamine abuse, presenting with persistent cholangiopathy [13]. The patient experienced recurrent abdominal pain, jaundice, and abnormal liver function tests. Imaging revealed bile duct strictures, and despite initial conservative treatments, the patient ultimately required endoscopic retrograde cholangiopancreatography (ERCP) and stenting to manage recurring cholangitis. Further extending the temporal scope of research, a systematic review covering studies from 1950 to 2023 identified 185 articles linking chronic ketamine use to a wide array of side effects, including impaired verbal processing, cystitis, and cholangiopathy [14]. The review emphasized the prevalence of gastrointestinal (GI) symptoms, particularly abdominal pain and vomiting, in patients with ketamine-associated cholangiopathy, reinforcing the notion that ketamine can trigger substantial hepatobiliary dysfunction, often resembling GI pathologies. Wong et al. provided further clinical insight through a case series documenting three patients with over a year of ketamine abuse [15]. These patients presented with a combination of urinary symptoms and cholestasis, along with fusiform dilation of the bile duct, which mimicked the appearance of a choledochal cyst. These rare presentations emphasize the diverse clinical manifestations of ketamine toxicity, which may extend beyond typical hepatobiliary symptoms. Building on these findings, a case series documented six young adults with chronic ketamine use, all of whom developed severe cholestasis and cholangiopathy [16]. These patients presented with abdominal pain and biliary duct dilation suggestive of sclerosing cholangitis. Another individual case report discussed that a patient’s alkaline phosphatase, gamma-glutamyl transferase, and alanine transaminase levels were significantly elevated at 970 IU/L, 1796 IU/L, and 46 IU/L, respectively, during the acute phase of ketamine abuse [17]. However, these values decreased to near-normal levels after 60 days of abstinence and appropriate medical management, demonstrating the potential for biochemical recovery once ketamine use ceases. Further supporting the link between ketamine use and biliary damage, Yu et al. reported fusiform dilation of the common bile duct (CBD) with smooth distal tapering as the most common imaging feature in ketamine-induced cholangiopathy [18]. These radiologic findings are pivotal for clinicians, as they provide diagnostic confirmation for identifying ketamine-related biliary abnormalities. Another noteworthy study described three young males with chronic ketamine use, each presenting with cholestasis and biliary tract abnormalities [19]. This study highlighted fluctuating liver function and dilated biliary systems on CT imaging, with one patient exhibiting elevated bilirubin levels exceeding 3 µmol/L. This fluctuation in liver biochemistry underscores that ketamine-related liver dysfunction can be of an episodic nature as well. In addition to these individual case reports and series, a retrospective study involving 37 chronic ketamine users revealed that 73% of participants experienced upper GI symptoms, predominantly epigastric pain [20]. Endoscopic evaluations showed that 85.7% of these patients had gastritis. Interestingly, the cessation of ketamine use led to a significant improvement in symptoms, with a p-value of 0.027 and an odds ratio (OR) of 12.5 (95% CI 1.20, 130.6), indicating a strong association between ketamine cessation and symptom relief. A cross-sectional survey of 297 chronic ketamine abusers with urinary tract dysfunction identified liver injury in 9.8% of the cases, all of which were classified as cholestatic [21]. Liver biopsies performed on seven patients revealed bile duct injury, and in two cases, there was evidence of bridging fibrosis. Additionally, imaging of six patients indicated CBD dilation in three cases, though no obstructions were found. This large-scale study adds further weight to the link between chronic ketamine use and liver injury, particularly in the context of co-occurring urinary tract dysfunction. The results of the systematic reviews are summarized in Table 1, while the findings from other studies are compiled in Table 2. 

**Table 1:** Key findings and characteristics of systematic reviews NMDA-R: N-methyl-D-aspartate receptor, LFT: liver function test.

Study	Year	Country	Study type	Articles screened	Articles included	Epidemiology	Pharmacokinetics	Pharmacodynamics	CYP450 system effects	Acute side effects	Chronic side effects	Patient outcomes	Primary endpoint (PE)	Secondary endpoint (SE)	Conclusion
Teymouri et al. [12]	2024	Iran	Systematic review	4,512	143	Increasing prevalence of ketamine-induced biliary disease, mostly in chronic users (avg. use 3-15 years). High risk in males and those with co-existing alcohol abuse	Hepatic metabolism, biliary excretion, delayed clearance in chronic users	NMDA-R antagonist also affects opioid and dopamine pathways. Long-term use linked to persistent LFT abnormalities	Chronic ketamine exposure leads to CYP3A4 and CYP2C9 upregulation, resulting in increased liver stress and cholestasis	GI distress, nausea, jaundice, and transient LFT elevation	Persistent biliary strictures, liver fibrosis, increased risk of cholangiocarcinoma (rare but reported)	Approximately 88% improved after stopping ketamine, 12% had persistent cholangiopathy requiring intervention	Characterizing biliary complications in ketamine abusers	Correlation between disease severity and duration of ketamine use	Ketamine-induced biliary dysfunction can persist even after drug cessation. Early diagnosis and intervention are crucial
Schep et al. [14]	2023	New Zealand	Systematic review	5,268	185	Ketamine use is increasing, particularly in the dance/rave scene and among recreational drug users in the US, Europe, and Asia. High prevalence in young adults (18-35 years)	Rapid absorption, metabolized extensively in the liver by CYP enzymes, half-life 1.5-5 hours, primarily eliminated via kidneys	NMDA receptor antagonist, induces dissociative anesthesia, maintains respiratory drive, causes hallucinations at low doses	Metabolized by CYP2B6, CYP3A4, CYP2C9; converted to norketamine → hydroxynorketamine and dehydronorketamine; affecting metabolism and liver function	Hallucinations, dizziness, irrational behavior, vomiting, abdominal pain, tachycardia, hypertension	Cholangiopathy, cystitis, cognitive impairment, hepatobiliary dysfunction, recurrent GI issues, increased risk of depression	Variable: some improve with abstinence, others developed chronic liver or bladder damage	Characterize the toxicity, metabolism, and long-term effects of ketamine	Understand the role of CYP enzymes in ketamine metabolism and its impact on the hepatobiliary system	Ketamine induces liver and biliary dysfunction, affecting CYP metabolism. More research is needed on long-term effects and treatment strategies

**Table 2:** Key findings and characteristics of other studies included MRCP: magnetic resonance cholangiopancreatography, US: ultrasound, CT: computed tomography, ERCP: endoscopic retrograde cholangiopancreatography, CBD: common bile duct, LFT: liver function test, GI: gastrointestinal.

Study	Year	Country	Study design	No. of patients	Mean age ± SD	Male (%)	Female (%)	Common symptoms	Imaging modality and findings	Significant findings	Primary endpoint (PE)	Secondary endpoint (SE)	Treatment	Outcome (% improved, worsened, recurrence)
Nyirenda et al. [13]	2020	United Kingdom	Case report	1	32	100%	0%	Jaundice, cholangitis, rigors	MRCP: diffuse mural irregularity of bile ducts, recurrent cholangitis	Liver dysfunction progressed despite ketamine cessation	Effectiveness of biliary stenting in ketamine-induced cholangitis	Long-term outcomes after stenting	Biliary stenting, supportive care	0% improved, 100% recurrence
Wong et al. [15]	2009	Hong Kong	Case series	3	24 ± 3	50%	50%	Recurrent epigastric pain	US, CT, ERCP, MRCP: CBD dilation mimicking choledochal cyst	Symptoms correlated with ketamine use; ERCP showed smooth tapered CBD dilation	Relationship between ketamine abuse and CBD dilation	Effects of ketamine cessation on imaging findings	Nasobiliary drain, conservative	67% improved, 33% recurrence
Garkusha et al. [16]	2024	United States	Case series	6	26 ± 6.2	50%	50%	Abdominal pain, nausea, weight loss, urinary symptoms	US, MRCP, EUS: CBD dilation up to 17 mm, sclerosing cholangitis features	LFT abnormalities persisted in some cases	Identifying sclerosing cholangitis-like changes in ketamine users	Differences in disease progression between males and females	Supportive, ERCP, biliary drainage	50% improved, 33% worsened, 17% recurrence
Pappachan et al. [17]	2014	United Kingdom	Case report	1	59	100%	0%	Cachexia, hepatobiliary dysfunction, renal failure	US, CT, MRCP: Mild CBD dilation, hepatobiliary dysfunction, periportal hyperechogenicity	Multisystem involvement: GI, liver, kidney, cachexia	Multisystem effects of chronic ketamine abuse	Impact of nutritional status on recovery outcomes	Hydration, drug rehab	100% improved
Yu et al. [18]	2014	Hong Kong	Retrospective	26	31 ± 4.1	42.30%	57.70%	Abdominal pain	CT, MRCP: Fusiform CBD dilation (69%), non-dilated intrahepatic ducts	Severity of dilation correlated with duration of ketamine abuse	Prevalence of ketamine-related cholangiopathy	Reversibility of imaging findings with abstinence	Abstinence, supportive care	73% improved, 27% worsened
Lo et al. [19]	2011	United Kingdom	Case series	3	27 ± 2.5	100%	0%	Obstructive jaundice	US, MRCP, ERCP: Biliary dilatation up to 1.4 cm	Ketamine metabolite detected in urine; recurrent biliary symptoms post-exposure	Clinical course of ketamine-induced biliary obstruction	Effectiveness of ERCP in symptom relief	ERCP, stenting	0% improved, 100% recurrence
Poon et al. [20]	2010	Hong Kong	Retrospective	37	25 ± 3.6	67.20%	32.80%	Epigastric pain	Endoscopy: gastritis, normal endoscopy in some cases	Abstinence significantly associated with symptom relief (OR 12.5)	Association between ketamine use and upper GI symptoms	Effect of *H. pylori*-negative gastritis on outcomes	Abstinence	92% improved, 8% no improvement
Wong et al. [21]	2014	Hong Kong	Cross-sectional	297	25 ± 4	47%	53%	Hepatobiliary dysfunction	MRCP, US, biopsy: CBD dilation, fibrosis, bile duct injury	9.8% had liver injury; bile duct injury observed in all biopsied cases; 2 had bridging fibrosis	Histopathological features of ketamine-related liver injury	Gender differences in risk of fibrosis	Supportive, monitoring	91% improved, 9% worsened

Discussion

Our literature review highlights that while ketamine is beneficial for pain management, sedation, and emerging treatments in psychiatric conditions, it is also associated with significant hepatobiliary side effects. The evidence collected underscores a range of hepatobiliary issues linked to ketamine use, including cholestasis, biliary duct abnormalities, and liver enzyme elevations. Understanding the pathophysiology of ketamine-induced liver and hepatobiliary injury is crucial, as it will serve as the cornerstone for developing future guidelines for treatment, prevention, and safer use of ketamine in clinical settings. The exact mechanisms of ketamine-induced hepatobiliary injury remain unknown, though several hypotheses exist in the literature. One prevailing hypothesis is that ketamine influences the activity of NMDA ionotropic receptors located on smooth muscle in the biliary tract [22]. It has been postulated that ketamine abuse leads to bile duct injury, cholestasis, and CBD dilation, either through a direct effect on biliary smooth muscle or via central actions [23]. Another potential mechanism involves the disruption of gallbladder motility through central pathways [24]. Additionally, ketamine has been found to induce oxidative stress and apoptosis in hepatocytes, further contributing to hepatic damage [25]. Since ketamine is metabolized by the cytochrome P450 system in the liver and excreted in bile and urine, direct toxic injury to the surface epithelium has also been suggested as a plausible mechanism [13]. The association between elevated C-reactive protein levels and abnormal liver biochemistry suggests that a chronic inflammatory process may play a role. However, despite the presence of multiple hypotheses, it is clear that research in this area is limited. Expanding research on ketamine's effects on other organs, such as the urinary tract, could also provide valuable insights into the biochemical mechanisms behind its hepatobiliary toxicity. The clinical implications of ketamine-induced liver injury emphasize the need for comprehensive liver assessments in patients using ketamine, whether for recreational, medical, or surgical purposes. Managing ketamine-induced hepatobiliary damage effectively requires a multifaceted approach that addresses both the immediate and long-term consequences of liver injury. In the acute phase, immediate cessation of ketamine is essential to prevent further hepatic damage. Supportive care, including fluid administration, electrolyte replacement, and close monitoring of liver function, plays a vital role in stabilizing the patient's condition. For patients with more severe or persistent liver damage, pharmacological interventions may be necessary. Ursodeoxycholic acid, a bile acid that improves bile flow and reduces inflammation, has shown potential in managing ketamine-induced cholestatic liver injury [25]. In addition to pharmacological treatments, lifestyle modifications and strict adherence to abstinence from ketamine are critical for long-term management. Educating patients about the importance of a healthy diet, avoiding alcohol and other hepatotoxic substances, and maintaining a regular exercise routine can support liver regeneration and overall health [26]. In conclusion, ketamine abuse can lead to a range of liver injuries, from CBD dilation to bile duct damage and even significant liver fibrosis. Further research is necessary to explore the natural history, underlying mechanisms, and potential therapeutic targets for ketamine-induced hepatobiliary injuries.

Limitations

This review has several limitations, including a limited number of studies that restrict the generalizability of findings and significant heterogeneity in study designs, patient populations, and reported outcomes, which complicates comparisons. The reliance on case reports and series introduces potential biases and may not fully represent the range of ketamine's hepatobiliary effects. The exclusion of non-English language studies and lack of long-term follow-up data may result in an incomplete understanding of ketamine's impact on the liver. Additionally, confounding factors such as polypharmacy and pre-existing liver conditions were not consistently accounted for, potentially affecting the interpretation of the results.

## Conclusions

This review underscores the potential for significant hepatobiliary toxicity associated with ketamine use, ranging from mild liver enzyme elevation to severe cholestasis and biliary tract abnormalities. While ketamine's therapeutic benefits are notable, its safety profile requires careful consideration. The findings suggest a need for further research to clarify the underlying mechanisms, establish risk factors, and develop strategies to mitigate adverse effects. Clinicians should remain vigilant in monitoring liver function in patients receiving ketamine, ensuring informed decision-making and safe clinical use.
